# Dietary oyster mushroom fermented *Vachellia erioloba* pods enhance Boschveld chicken meat healthiness without altering its physicochemical quality, growth performance and physiology

**DOI:** 10.1038/s41598-024-77142-x

**Published:** 2024-10-29

**Authors:** Melokuhle Q. Magagula, Makiwa S. Mthana, Doctor M. N. Mthiyane

**Affiliations:** 1 Department of Animal Science, School of Agricultural Sciences, Faculty of Natural and Agricultural Sciences, North-West University (Mahikeng Campus), Private Bag X 2046, Mmabatho, 2735 South Africa; 2 Food Security and Safety Focus Area, Faculty of Natural and Agricultural Sciences, North-West University (Mahikeng Campus), Mmabatho, 2735 South Africa

**Keywords:** Indigenous chickens, Meat quality, Fatty acids, Nutritional status, *Vachellia erioloba* pods spent substrate, Biotechnology, Physiology

## Abstract

The high content of fibre and antinutritional phytochemicals limit the utilization of *Vachellia erioloba* tree pods as nutraceutical feed additive for indigenous chicken diets. The pods can however be solid-state fermented using oyster mushrooms to enhance the nutritional utility of their spent substrate for the nutrition of the native birds. Therefore, this study investigated the effects of dietary incorporation of *V. erioloba* pods oyster mushroom spent substrate (OMSS) on growth performance, carcass traits, visceral organs, haemato-biochemistry, and meat quality including its fatty acid composition in Boschveld chickens. In a completely randomized design, 250 4-week old mixed gender Boschveld chicks were randomly allotted to 25 pens in which they were offered treatment diets (0, 1.25, 2.5, 5 and 10% OMSS) each with 5 replicates of 10 for 12 weeks and then slaughtered. While there were neither linear nor quadratic effects of diet on overall feed intake (FI) (*P* > 0.05) and body weight gain (BWG) (*P* > 0.05), dietary incorporation of OMSS decreased overall feed conversion efficiency (FCE) (quadratic: *P* < 0.05) particularly in weeks 5 (linear: *P* < 0.05), 6 (quadratic: *P* < 0.01) and 11 (quadratic: *P* < 0.05) with no effects in subsequent weeks (*P* > 0.05). Also, OMSS induced no effects on all carcass characteristics, visceral organs, haemato-biochemistry and meat physico-chemical quality (*P* > 0.05) except for the increase in serum albumin (quadratic: *P* < 0.05) and bilirubin (quadratic: *P* < 0.05) as well as 24 h post-slaughter meat lightness (linear: *P <* 0.01), redness (quadratic: *P <* 0.05), yellowness (linear: *P <* 0.05), hue angle (quadratic: *P <* 0.05), and drip loss (quadratic: *P <* 0.05). Further, the spent substrate decreased meat myristic (linear: *P* < 0.01), palmitic (linear: *P* < 0.05), palmitoleic (linear: *P* < 0.01), and oleic (linear: *P* < 0.01) acids, as well as its total polyunsaturated fatty acids (PUFAs) (linear: *P* < 0.05), monounsaturated FAs (MUFAs) (quadratic: *P* < 0.01), and *n*-6 PUFAs (linear: *P* < 0.05). Furthermore, it decreased the meat *n*-6/*n*-3 PUFA ratio (quadratic: *P* < 0.01), with meat from birds fed diets incorporated with 2.5% OMSS eliciting the lowest ratio of 3.63. In contrast, dietary OMSS increased meat stearic (linear: *P* < 0.001), docosahexaenoic (quadratic: *P* < 0.01), and tricosanoic (linear: *P* < 0.001) acid concentrations as well as its total saturated FAs (SFAs) (linear: *P* < 0.01) and *n*-3 PUFAs (quadratic: *P* < 0.01). In conclusion, dietary feeding of *V. erioloba* pods-derived OMSS enhanced meat nutritional healthiness without majorly altering its physico-chemical quality as well as growth performance, carcass traits, and haemato-biochemistry in Boschveld indigenous chickens. It is recommended for inclusion in indigenous chicken diets at 2.5% level.

## Introduction

The rapidly burgeoning human population of South Africa has escalated the severity of food insecurity and heightened the urgency for improved agricultural production to address the national food insecurity challenges particularly among the rural and township-based inhabitants^1^. With almost 90% of households in the country keeping indigenous chickens (*Gallus domesticus*) under traditional free range semi-scavenging smallholder systems^2^, improved production of these birds can help cascade many households out of food insecurity. Produced intensively and sustainably, the native chickens, among which is the Boschveld, can also help improve the socio-economic status of local communities as a source of income^3–5^. However, their productivity is currently low consequent to their scavenged feed materials-derived inadequate nutrition that is only sufficient for maintenance^6,7^. Worse, most smallholder farmers who rear them cannot afford expensive commercial diets based on maize and soyabean meal^8,9^. This accounts for the burgeoning interest in exploration of cheaper locally abundant yet underutilized alternative feed resources such as *V. erioloba* pods for the native birds.

The remarkably large, light-grey, indehiscent, and crescent moon-shaped velvety seed pods of *V. erioloba* trees [formerly *Acacia erioloba*; *Fabaceae* family; common name: Black-barked Camel (Giraffe) Thorn]^10^ can be a good source of nutrition for indigenous chickens. They annually become abundantly available for consumption by ungulates including elephants mainly during the autumn and winter seasons^11^. Otherwise, they are powdered and used by local people for treatment of ear infections whereas the strong wood of the tree is used as firewood^12^, the roots for treatment of tooth aches and tuberculosis upon boiling and gargling of the infusion, and the seeds are roasted and used as coffee bean replacement^13^. The pod-producing trees are widespread in arid and semi-arid northern provinces of South Africa including parts of Free State, North West, Northern Cape, Gauteng, Limpopo, and Mpumalanga as well as other southern African countries including Botswana, Namibia, Eswatini, Angola, Zambia and Zimbabwe^14^. Produced by the *V. erioloba* trees with the deepest growing roots of any known species, with a recorded maximum of 68 m, that allow them access to deep groundwater sources, the pods are most ideal for use as animal feed in this epoch of climate change and recurrent droughts with concomitant shortages and high costs of feeds^15^.

Nutritionally, the seed-bearing pods are a good source of protein (124.1 g/kg DM); amino acids particularly aspartic acid, glutamic acid, proline and tyrosine; energy (19.0 MJ/kg, similarly to maize); minerals particularly iron, zinc, manganese and calcium; as well as vitamins^16,17^. Their major limitation, however, is their high content of fibre (362.0 g/kg DM)^16,18^ and antinutritional factors (ANFs) in the form of the deadly prussic acid-convertible cyanogenic glycosides particularly in green pods^19,20^, as well as tannins (145.4 g/kg DM), protease inhibitors and others^21^. This therefore renders the pods an ideal substrate for solid-state fermentation (SSF) to enhance their utility as a feed resource for indigenous chickens. Generally, SSF decreases the concentrations of fibre and ANFs in plant-derived feed resources whilst enhancing their nutraceutical composition particularly in terms of protein (amino acids and enzymes), minerals, as well as antioxidant and antimicrobial compounds, leading to improved growth performance in chickens fed diets incorporated with the spent substrate^22,23^. In this regard, mushrooms are very effective as solid-state fermenters. The mycelium of oyster mushrooms (*Pleurotus ostreatus*) secretes bountiful amounts of ligninolytic enzymes particularly effective in biodegrading fibre including the recalcitrant lignin as well as various noxious compounds transforming them into non-toxic or less toxic molecules during SSF^24^. Therefore, the objective of this study was to investigate effects of dietary incorporation of oyster mushroom-fermented *V. erioloba* pods (OMSS) on growth performance, carcass characteristics, haemato-biochemistry, as well as meat physico-chemical quality including fatty acid composition, of Boschveld indigenous chickens.

## Materials and methods

### Animal ethics statement

All experimental protocols were approved by the Animal Production Research Ethics Committee of North-West University (NWU) (Ethics Number: NWU-00804-23-A5). All methods were carried out in accordance with the ARRIVE guidelines (https://arriveguidelines.org/).

### Study site and ingredient sources

The study was carried out at NWU Experimental Farm at Molelwane located ~ 8 km away from Mahikeng central business district in the North West Province of South Africa. *V. erioloba* pods were harvested at the same experimental farm while all feed ingredients were provided by SimpleGrow Agric Services (Pty) Ltd (Centurion, Gauteng Province, South Africa) and the oyster mushroom spawn was supplied by Eco-Agro Enterprise (Pty) Ltd (Mbombela, Mpumalanga Province, South Africa).

### Oyster mushroom spent substrate production

Prior to substrate preparation, *V. erioloba* pods were dried under shade and coarsely ground using a milling machine without a screen. The preparation of *P. ostreatus* spawn at Eco-Agro Enterprise (Pty) Ltd was as reported by Mhlongo and Mnisi^25^ whilst substrate inoculation was performed as described by Mthana and Mthiyane^26^, however with minor modifications. Briefly, the coarsely ground pods were soaked in distilled water for 4 h and sterilized at 120 ℃ for 1 h in an autoclave. The sterilized substrate was then inoculated in sterile mushroom bags with 500 g/kg of spawn. The bags were perforated and kept in a dark incubation room set at 25 ℃ and 75% relative humidity for 28 days. On day 29, the OMSS was then harvested, dried under shade, milled (1 mm screen size), and stored in polyethylene bags at room temperature pending chemical analysis.

### Chemical analysis

The proximate composition in terms of dry matter (method 930.15), crude protein (method 954.01), crude fibre (method 978.10), ether extract (method 920.39), and ash/organic matter (method 942.05) of un-fermented and fermented (OMSS) *V. erioloba* pods (Table 1) and experimental diets (Table 2) was analyzed using the AOAC^27^ methods. The same AOAC methods were also used for amino acid analysis (method 991.25) of experimental diets. Neutral detergent fibre (NDF) and acid detergent fibre (ADF) were analyzed using methods described by Van Soest et al.^28^ while acid detergent lignin (ADL) was determined by soaking the ADF residue bags in 72% H_2_SO_4_ for 3 h and oven-drying them for 6 h at 105 ℃. The minerals were analyzed using an Inductively Coupled Plasma Mass Spectrometer (ICP-MS) [NexION^®^ 2000, PerkinElmer (Pty) Ltd] after sample digestion using an automated sample digester [Titan Microwave Sample Preparation System, PerkinElmer (Pty) Ltd]. Total phenolics were determined following the method described by Makkar et al.^29^, whereas total tannins were quantified using the procedure described by Porter et al.^30^. Hydrogen cyanide (HCN) was measured using a modified method of Dahal and Tamang^31^ that employs endogenous linamarase to hydrolyse linamarin to release free cyanide that is then quantified by colorimetry in picrate solution. Absorbance was then measured at 535 nm and the HCN concentration calculated as: *HCN* = (*Y* × 50 × 1000) / (5 × *sample weight*), where *Y* = 0.0304x + 0.0054 (R^2^ = 0.969) and x = absorbance. Metabolizable energy (ME) was calculated according to Livesey^32^ as ME (kcal/kg) = 2778–66 (CF × 88%DM) and then converted into MJ/kg.

**Table 1:** The nutritional composition (%, on DM basis, unless stated otherwise) of unfermented and oyster mushroom-fermented spent substrate of *Vachellia erioloba* pods.

Parameter	Unfermented	Fermented (OMSS)
Dry matter	92.02	92.90
Crude protein	14.2	23.69
Crude fibre	36.06	24.88
Ether extract	1.6	2.30
Ash	4.3	11.61
Metabolizable energy (MJ/kg)	8.54	10.32
Neutral detergent fibre	46.8	32.34
Acid detergent fibre	32.1	19.78
Calcium	0.67	1.02
Phosphorus	0.13	0.25
Potassium	1.4	2.66
Sodium	0.01	0.43
Hydrogen cyanide (mg/100kg)	22.5	6.25
Condensed tannins (AU_550nm_/10 mg DM)	0.73	0.22
Total phenolics (GA equivalent µg/mg DM)	5.66	1.51

AU = absorbance units; DM = dry matter; GA = gallic acid.

**Table 2:** Ingredient and nutritional composition (%, on as-fed basis) of experimental diets for Boscveld indigenous chickens.

Ingredient	Dietary OMSS inclusion level (%)				
	0	1.25	2.5	5	10
Yellow maize fine	59.00	59.00	59.00	60.00	59.28
Soyabean meal (46.5% CP)	18.67	18.23	18.23	21.53	18.19
Wheat bran	8.36	7.32	8.95	6.32	8.03
Sunflower oilcake (34% CP)	7.00	7.00	0.00	0.00	0.00
Crude soya oil	2.45	2.65	2.65	2.65	0.00
Feed lime fine	1.50	1.50	1.50	1.50	1.50
Pellibond	1.00	1.00	1.00	1.00	1.00
OMSS	0.00	1.25	2.50	5.00	10.00
Mono-dicalcium phosphate	0.72	0.72	0.70	0.70	0.70
L-Lysine HCL	0.15	0.17	0.16	0.16	0.16
DL-Methionine	0.24	0.25	0.24	0.24	0.24
L-Threonine	0.15	0.16	0.15	0.15	0.15
Salt fine	0.20	0.20	0.20	0.20	0.20
Sodium bicarbonate	0.22	0.20	0.19	0.19	0.19
Avatec 15% (Lasolacid 15%)	0.05	0.05	0.05	0.05	0.05
Zinc bacitracin 15%	0.05	0.05	0.05	0.05	0.05
AxtraPhytase Broiler (1000FTU)	0.01	0.01	0.01	0.01	0.01
*Broiler grower premix	0.25	0.25	0.25	0.25	0.25
Total	100	100	100	100	100
*Analysed chemical composition*					
Dry matter	91.79	91.97	92.00	91.46	91.75
Crude protein	17.48	16.66	16.84	16.97	16.88
Crude fibre	5.46	5.13	5.81	5.44	5.58
Ash	7.32	5.15	5.18	5.45	4.77
Ether extract	6.48	6.36	6.48	7.61	7.22
Neutral detergent fibre	17.05	17.20	16.62	16.80	17.75
Acid detergent fibre	7.59	8.63	6.67	7.39	9.30
Acid detergent lignin	2.08	3.00	2.40	2.82	2.17
Metabolizable energy (MJ/kg)	11.50	11.50	11.50	11.50	11.50
Lysine	0.80	0.80	0.84	0.81	0.73
Methionine	0.52	0.50	0.47	0.46	0.45
Threonine	0.68	0.68	0.67	0.65	0.60
Calcium	0.70	1.10	0.92	1.06	0.94
Phosphorus	0.63	0.63	0.57	0.60	0.56
Potassium	0.83	0.80	0.81	0.76	0.71
Sodium	0.18	0.18	0.18	0.19	0.21
Chloride	0.21	0.21	0.20	0.20	0.20

*Broiler grower premix: 0.12 g biotin; 8.0 mg copper sulphate; 80 mg ferrous sulphate; 0.7 mg folic acid; 79 mg zinc sulphate; 30 mg niacin; 100 mg magnesium sulphate; 0.34 mg potassium iodine; 10 mg pantothenic acid; 2.5 mg vitamin B1; 5.1 mg vitamin B6; 2500 IU vitamin D3; 2.0 mg vitamin K3; 4.5 mg vitamin B2; 25 IU vitamin E; and 0.25 mg sodium selenite; 11 000 IU vitamin A; OMSS = oyster mushroom spent substrate.

### Diet formulation, experimental design and performance measurements

Five maize-soyabean meal-based grower diets were formulated to meet nutritional requirements of indigenous chickens. The diets were formulated such that they contained graded levels (0, 1.25, 2.5, 5, and 10%) of OMSS (Table 2). Then, using a random number table, a total of 250 4-weeks-old Boschveld indigenous chicks with homogeneous initial body weights (230.96 ± 3.8) were randomly and evenly assigned to 5 dietary treatments in a randomized complete block design (RCBD) for 12 weeks (weeks 5 to 16). Each treatment had 5 replicate pens (dimensions: 3.5 m long × 1.0 m wide × 1.85 m high) with 10 birds (5 roosters and 5 hens) each. Then, the 25 experimental pens were divided into 5 blocks of 5 consecutive pens aimed at capturing spatial variation within the chicken house. Within each block, the pens were randomly assigned to 1 of 5 experimental dietary treatments. The birds had free access to feed and fresh water under natural lighting conditions during the day (8:00 am to 17:00 pm) and with electric lighting at night (18:00 pm to 07:00 am) throughout the experimental period. Feed intake (FI) was then calculated as the difference between feed offered and feed refusals divided by the total number of birds per pen. Then weekly FI was calculated by combining the daily FI values over a period of 7 days. The body weight gain (BWG) was calculated as the difference between the current and previous body weight divided by the total number of birds per pen. Feed conversion efficiency (FCE) was ultimately calculated by dividing the BWG with FI.

### Blood collection and analysis

At the terminal stage of the experiment, 2 birds (1 rooster and 1 hen) were randomly selected from each pen for blood collection. Approximately 5 mL of blood was collected from the wing vein of each bird and 1 mL was placed into purple-topped vacutainers coated with EDTA for haematology analysis using the IDEXX LaserCyte Haematology Analyzer (IDEXX Laboratories (Pty) Ltd., Johannesburg, South Africa). On the other hand, 4 mL of blood was placed into red-topped vacutainers without anticoagulant for serum biochemical analysis. The collected blood was centrifuged (3500 rpm, 4 °C) for 10 min to obtain serum, which was used for biochemical analysis using a computerized IDEXX Vet Test Chemistry Analyzer (IDEXX Laboratories (Pty) Ltd., Johannesburg, South Africa).

### Slaughter, visceral organs, and carcass traits measurements

In the evening of the day before slaughter, all birds were deprived of feed for 12 h while having access to drinking water. Next morning, they were group-weighed to obtain slaughter weights. They were then immediately transported for slaughter to the nearest poultry abattoir in Rooigrond about 30 km away from the experimental site. At the abattoir, they were electrically stunned (70 volts), humanely sacrificed by cervical dislocation, slaughtered by cutting the jugular vein with a sharp knife, and allowed to bleed for 5 min. The feathers, head, neck, and feet were removed and then all visceral organs manually pulled out through an incision from the vent to the sternum. Then, carcasses were washed and weighed to obtain hot carcass weights followed by chilling for 24 h at 4 ℃ and reweighing to attain cold carcass weights. Afterward, the spleen, liver, gizzard, duodenum, jejunum, ileum, caecum, and colon were individually weighed and expressed as relative percentage of hot carcass weight. The carcass cuts (breast, wing, thigh, and drumstick) were also individually weighed, and their weights expressed as relative percentage of CCW.

### Meat physico-chemical analysis

Meat physico-chemical analysis was determined on the left side of the breast muscle from 3 meat samples per replicate pen (15 per treatment). The meat pH and colour [lightness (L*), redness (a*), and yellowness (b*)] were measured 45 min and 24 h after slaughter. Meat pH and temperature were measured using a portable digital pH-temp meter (Hanna Instruments, model HI98163, Johannesburg, South Africa) equipped with a silver spear-type electrode. The pH meter was calibrated using standard buffers of pH 4, 7, and 10 before analysis and after every batch of 10 samples. Meat colour was measured using a meat colour guide (Konica Minolta CR-400, Narich, Japan) with a 20 mm diameter measurement area. Chroma (C*) and hue angle (H*) values were calculated following methods described by the American Meat Science Association (AMSA)^33^. A filter paper pressure-press method described by Grau and Hamm^34^ was used to determine the water holding capacity (WHC). Drip loss was determined using the method described by Honikel^35^ where meat samples (~ 8 g) were hooked inside sample bottles and stored in a chiller (4 ℃) for 72 h. The samples were hooked such that they did not get in contact with the surface or sides of the bottle. For cooking loss measurement, pre-weighed meat samples were oven-cooked at 75 ℃ for 20 min, water discarded, and samples allowed to cool at room temperature and re-weighed. Thereafter, the cooked meat was sheared perpendicular to muscle fibres using a Meullenet-Owens razor shear blade attached to a Texture Analyzer (TA-XT plus, Stable Micro Systems, Surrey, UK) to determine the meat shear force.

### Meat fatty acid analysis

Total lipids were quantitatively extracted from 3 meat samples per replicate pen (15 per treatment) using the method outlined by Folch et al.^36^ that uses chloroform and methanol in a ratio of 2:1 with hendecanoic acid (C11:0) as internal standard (following establishment that it was undetectable in the study samples). Fatty acid methyl esters (FAMEs) were prepared for gas chromatography by methylation of the extracted fat using methanol–BF3 on an Agilent 7890 A GC system equipped with a 100 m × 0.25 mm ID (0.20 μm film thickness) Supelco SP-2560 capillary column (Supelco, Inc., Bellefonte, PA, USA). Then, 1 µL of FAME was injected into the chromatograph with a flame ionization detector, using helium as the carrier gas and a split ratio of 10:1 post-injection. The injector temperature was programmed to 250 ℃ and the detector temperature to 300 ℃. The column temperature program began at 120 ℃ for 5 min, increased by 2 ℃/min to 170 ℃, held at 170 ℃ for 15 min, increased by 5 ℃/min to 200 °C, held at 200 °C for 5 min, increased by 2 ℃/min to a final temperature of 235 ℃, and held for 10 min. Fatty acid concentrations were expressed as a percentage of total identified fatty acids. The obtained fatty acid data were used to calculate the totals of saturated (SFAs), mono-unsaturated (MUFAs), and poly-unsaturated (PUFAs) fatty acids, as well as *omega*-6 (*n*-6) PUFAs, *omega*-3 (*n*-3) PUFAs, and the *n*-6/*n*-3 PUFA ratio.

### Statistical analysis

Prior to statistical analysis, all data were evaluated for normality using Proc Univariate in the Statistical Analysis System (SAS)^37^ version 9.4. Normally distributed data with homogenous variances were subjected to One-Way ANOVA in the general linear model (GLM) procedure of SAS and the least square means (LSMeans) separated using the probability of difference (PDIFF) option within the LSMeans statement, with significance determined at *P* ≤ 0.05. No block effect on overall performance and all other responses of birds was observed and therefore the block factor was not used in the statistical models but only the dietary treatment was considered a fixed factor. Dose-depended responses of the chickens to increasing dietary levels of OMSS were evaluated using polynomial contrasts (RSREG PROC, SAS^37^). However, for weekly FI, BWG and FCE, the data were analysed as repeated measures with dietary treatment and week as fixed factors using the statistical model: y_*ijk*_ = µ + α_*i*_ + β_*j*_ +(αβ)_*ij*_ +ε_*ijk*,_ where y_*ijk*_ = response variable, µ = overall mean, α_*i*_ = effect of diet, β_*j*_ = effect of week, (αβ)_*ij*_ = diet × week interaction effect, and ε_*ijk*_ = random error.

### Animal welfare statement

The authors certify that they have complied with the journal’s ethical policies, as stated on the author guidelines page, and that they have obtained the necessary approval from the ethical review committee. The authors affirm that they complied with EU regulations on the care of animals used for research purposes.

### Research involving animals

The study was conducted following ARRIVE guidelines.

## Results

### Growth performance, carcass traits, visceral organ, and haemato-biochemistry

The effects of dietary incorporation of graded levels of OMSS on overall and weekly performance, carcass characteristics, visceral organ weights and lengths, and haemato-biochemistry of Boschveld chickens are presented in Tables 3, 4 and 5, respectively. While there were neither linear nor quadratic effects of dietary treatment on overall FI (*P* > 0.05) and BWG (*P* > 0.05), dietary inclusion of OMSS quadratically decreased overall FCE (*P* < 0.05) of the indigenous birds. Also, repeated measures analysis showed that whilst there was no diet × week interaction effects on FI (*P* > 0.05) and BWG (*P* > 0.05), there was a diet × week interaction effect on FCE (*P* < 0.01). In this connection, dietary OMSS decreased FCE in weeks 5 (linear: *P* < 0.05), 6 (quadratic: *P* < 0.01) and 11 (quadratic: *P* < 0.05) but induced no effects on this parameter in weeks 7, 8, 9, 10, 12, 13, 14, 15, and 16 (*P* > 0.05). Further, there were neither linear nor quadratic responses of all carcass characteristics (*P* > 0.05), visceral organs (*P* > 0.05), and haemato-biochemistry parameters (*P* > 0.05) to dietary OMSS, except for the spent substrate-induced quadratic increase in serum albumin (*P* < 0.05) and bilirubin (*P* < 0.05).

**Table 3:** Effects of dietary *Vachellia erioloba* pods oyster mushroom spent substrate inclusion on weekly and overall feed intake, body weight gain, and feed conversion efficiency of Boschveld indigenous chickens.

Parameter	Week	Dietary OMSS inclusion level (%)					SEM	*P*-value				
		0	1.25	2.5	5	10		Diet	Linear	Quadratic	Week	Diet × Week
FI (g/bird)	5	289.65	311.22	313.35	317.15	315.36	9.05	0.229	0.255	0.230	*P* < 0.001	0.934
	6	334.03^b^	355.43^ab^	373.30^a^	375.00^a^	359.09^ab^	10.73	0.086	0.401	*P* < 0.05		
	7	402.25^b^	431.92^ab^	449.10^a^	447.90^a^	460.25^a^	13.49	0.064	*P* < 0.05	0.172		
	8	467.80	490.40	487.20	493.60	473.50	11.93	0.489	0.646	0.171		
	9	483.10	526.70	496.80	510.50	503.10	13.53	0.261	0.884	0.465		
	10	530.20	583.70	547.90	571.22	562.00	15.33	0.162	0.516	0.299		
	11	538.10	568.50	553.30	550.40	561.60	17.35	0.776	0.712	0.979		
	12	561.90^b^	582.18^ab^	555.90^b^	573.10^ab^	600.00^a^	10.20	*P* < 0.05	*P* < 0.05	0.337		
	13	576.00	598.20	581.00	568.70	588.00	8.82	0.202	0.915	0.265		
	14	565.30	600.70	586.90	569.10	596.52	19.05	0.601	0.638	0.779		
	15	606.60	651.54	622.50	622.30	626.20	12.59	0.198	0.848	0.913		
	16	514.50	562.60	526.00	531.70	539.50	11.31	0.073	0.986	0.783		
BWG (g/bird)	5	110.80^b^	136.00^a^	120.00^ab^	121.60^ab^	111.60^b^	5.96	*P* < 0.05	0.278	0.241	*P* < 0.001	0.122
	6	116.73^c^	155.60^a^	149.60^ab^	146.00^b^	136.40^b^	19.25	*P* < 0.01	0.479	*P* < 0.05		
	7	161.02	93.64	115.20	123.60	110.40	18.60	0.167	0.733	0.815		
	8	132.80	140.76	143.60	138.80	143.60	9.67	0.928	0.679	0.871		
	9	142.80	178.40	156.00	186.00	168.80	11.46	0.096	0.329	0.123		
	10	113.60	125.60	120.00	124.80	109.20	14.54	0.469	0.513	0.746		
	11	134.40	165.60	149.60	160.80	104.00	15.16	0.061	0.062	0.058		
	12	102.00	130.00	136.80	118.00	106.80	17.59	0.596	0.653	0.285		
	13	100.40	93.60	104.00	92.00	94.00	13.73	0.965	0.704	0.877		
	14	117.20	142.00	144.40	104.80	124.40	15.84	0.376	0.487	0.671		
	15	98.40	125.20	122.40	111.20	97.60	10.01	0.196	0.204	0.226		
	16	108.80	114.40	96.80	94.80	125.20	20.31	0.816	0.690	0.234		
FCE (gain/feed)	5	0.38^b^	0.44^a^	0.38^b^	0.38^b^	0.35^b^	0.02	*P* < 0.05	*P* < 0.05	0.429	*P* < 0.001	*P* < 0.01
	6	0.28^b^	0.44^a^	0.40^a^	0.40^a^	0.38^a^	0.04	*P* < 0.001	0.557	0.065		
	7	0.41	0.21	0.26	0.28	0.24	0.05	0.071	0.400	0.585		
	8	0.28	0.12	0.30	0.28	0.30	0.01	0.921	0.526	0.744		
	9	0.30	0.34	0.31	0.36	0.34	0.02	0.268	0.333	0.183		
	10	0.21	0.16	0.16	0.22	0.19	0.02	0.349	0.596	0.931		
	11	0.25^ab^	0.29^a^	0.27^a^	0.29^a^	0.19^b^	0.02	*P* < 0.05	*P* < 0.05	*P* < 0.05		
	12	0.18	0.22	0.24	0.21	0.18	0.03	0.483	0.477	0.228		
	13	0.24	0.22	0.25	0.23	0.22	0.03	0.926	0.679	0.991		
	14	0.21	0.23	0.25	0.19	0.21	0.03	0.469	0.399	0.722		
	15	0.16	0.19	0.20	0.18	0.16	0.02	0.285	0.198	0.203		
	16	0.21	0.21	0.18	0.18	0.23	0.04	0.824	0.712	0.208		
Overall FI		5869.44	6263.09	6093.25	6130.67	6185.13	104.44	0.132	0.389	0.531		
Overall BWG		1399.60	1570.80	1528.40	1522.40	1432.00	46.08	0.082	0.369	0.082		
Overall FCE		0.24^a^	0.25^a^	0.25^a^	0.24^a^	0.23^b^	0.01	0.086	0.061	*P* < 0.05		

*FI* = feed intake, *BWG* = body weight gain, *FCE* = feed conversion efficiency, *OMSS* = oyster mushroom spent substrate, *SEM* = standard error of the mean.

**Table 4:** Effects of dietary *Vachellia erioloba* pods oyster mushroom spent substrate inclusion on carcass characteristics (% of CCW, unless stated otherwise) and visceral organs (% of HCW, unless stated otherwise) of Boschveld indigenous chickens.

Parameter	Dietary OMSS inclusion level (%)					SEM	*P*-value	
	0	1.25	2.5	5	10		Linear	Quadratic
*Carcass characteristics*								
Slaughter weight (g/bird)	1617.60	1818.80	1759.60	1758.00	1654.40	52.59	0.934	0.525
Hot carcass weight (g)	1302.00	1378.00	1284.00	1272.00	1272.00	58.26	0.398	0.783
Cold carcass weight (g)	1292.00	1368.00	1274.00	1260.00	1258.00	58.44	0.368	0.787
Dressing %	80.42	79.18	78.83	79.42	80.22	3.24	0.690	0.459
Breast weight (%)	11.79	11.13	12.17	10.38	11.19	0.42	0.226	0.275
Thigh weight (%)	8.88	8.54	7.79	9.12	8.95	0.44	0.438	0.634
Wing weight (%)	6.40	6.04	6.30	6.10	6.30	0.15	0.980	0.268
Drumstick weight (%)	7.92	8.04	7.93	7.64	7.93	0.27	0.772	0.479
*Visceral organs*								
Liver weight (%)	2.12	2.06	2.23	1.95	2.21	0.19	0.803	0.562
Gizzard weight (%)	2.35	2.23	2.03	2.08	2.26	0.14	0.851	0.079
Spleen weight (%)	0.30	0.31	0.37	0.31	0.36	0.05	0.434	0.934
Proventriculus weight (%)	0.60	0.48	0.55	0.51	0.49	0.05	0.248	0.594
Duodenum weight (%)	0.92	0.82	0.87	0.91	0.88	0.06	0.945	0.984
Jejunum weight (%)	1.24	1.06	1.21	1.36	1.20	0.11	0.674	0.428
Ileum weight (%)	1.24	1.06	1.27	1.19	1.07	0.12	0.471	0.643
Caecum weight (%)	0.86	0.78	0.81	0.93	0.81	0.07	0.966	0.434
Colon weight (%)	0.21	0.23	0.20	0.22	0.23	0.04	0.822	0.850
Duodenum length (cm)	16.85	19.82	20.63	16.75	19.70	2.70	0.796	0.983
Jejunum length (cm)	45.30	44.91	44.67	49.33	43.66	2.11	0.838	0.157
Ileum length (cm)	49.56	47.39	49.85	48.63	47.89	1.82	0.647	0.892
Caecum length (cm)	15.54	16.25	16.24	15.64	17.28	0.69	0.136	0.537
Colon length (cm)	5.99	5.78	6.31	6.76	6.41	0.37	0.207	0.230

*OMSS* = oyster mushroom spent substrate, *SEM* = standard error of the mean.

**Table 5:** Effects of dietary *Vachellia erioloba* pods oyster mushroom spent substrate inclusion on haematology and serum biochemistry of Boschveld indigenous chickens.

Parameter	Dietary OMSS inclusion level (%)					SEM	*P*-value	
	0	1.25	2.5	5	10		Linear	Quadratic
*Haematology*								
Red blood cells (×10^12^/L)	0.90	1.11	0.92	0.73	0.66	0.27	0.926	0.773
Haemoglobin (g/dL)	10.61	11.60	10.04	11.18	10.03	0.63	0.257	0.991
Reticulocytes (K/µL)	2.56	0.53	0.03	2.83	0.34	1.47	0.638	0.814
White blood cells (×10^9^/L)	141.37	62.22	103.49	152.04	124.02	32.12	0.699	0.733
Heterophils (K/µL)	4.32	4.00	4.58	5.11	6.34	1.84	0.567	0.664
Lymphocytes (×10^9^/L)	62.74	72.22	88.55	116.99	148.17	27.71	0.405	0.201
Monocytes (×10^9^/L)	49.71	44.97	49.44	45.01	38.97	14.84	0.799	0.987
Eosinophils (×10^9^/L)	3.51	2.66	2.21	2.21	1.75	0.78	0.214	0.952
Basophils (×10^9^/L)	0.09	0.06	0.04	0.02	0.05	0.02	0.344	0.585
Platelets (K/µL)	1599.75	1783.25	1743.17	1901.38	1651.50	275.23	0.446	0.128
Mean platelet volume (fL)	7.96	7.78	11.91	7.51	5.74	1.37	0.434	0.135
Platelet distribution width (%)	16.84	16.90	17.01	15.63	15.94	2.53	0.917	0.188
Procalcitonin (%)	1.44	1.91	0.79	1.43	1.37	0.30	0.356	0.823
*Serum biochemistry*								
Glucose (mmol/L)	11.13	11.68	10.86	11.28	11.94	0.48	0.278	0.485
Albumin (g/L)	16.00^a^	16.30^a^	15.90^ab^	15.70^ab^	15.50^b^	0.42	0.208	*P <* 0.05
Bilirubin (µmol/L)	4.00^ab^	4.10^a^	4.10^a^	3.70^ab^	3.50^b^	0.18	0.836	*P* < 0.05
Total protein (g/L)	38.60	39.20	36.70	40.70	37.60	2.26	0.877	0.622
Cholesterol (mmol/L)	3.31	3.18	3.04	2.89	3.16	0.16	0.534	0.954
Amylase(U/L)	305.80	354.30	380.24	362.63	394.40	54.31	0.935	0.731
Lipase (U/L)	8.40	7.60	12.70	9.90	9.00	2.47	0.909	0.380
Alkaline phosphatase (U/L)	825.20	733.30	926.80	854.40	875.60	83.42	0.501	0.674

OMSS = oyster mushroom spent substrate, RCDW = red cell distribution width, SEM = standard error of the mean.

### Meat quality parameters

The effects of dietary incorporation of graded levels of OMSS on breast meat quality and fatty acid composition of Boschveld chickens are, respectively, presented in Tables 6 and 7. Dietary OMSS induced neither linear nor quadratic effects on all meat physico-chemical quality parameters except for the increase in meat lightness (linear: *P <* 0.01), redness (quadratic: *P <* 0.05), yellowness (linear: *P <* 0.05), hue angle (quadratic: *P <* 0.05), and drip loss (quadratic: *P <* 0.05) only at 24 h post-slaughter. Also, dietary OMSS decreased meat myristic (linear: *P* < 0.01), palmitic (linear: *P* < 0.05), palmitoleic (linear: *P* < 0.01), and oleic (linear: *P* < 0.01) acids; totals of PUFAs (linear: *P* < 0.05), MUFAs (quadratic: *P* < 0.01), and *n*-6 PUFAs (linear: *P* < 0.05); as well as the *n*-6/*n*-3 PUFA ratio (quadratic: *P* < 0.01) as it increased its stearic (linear: *P* < 0.001), docosahexaenoic (DHA) (quadratic: *P* < 0.01), and tricosanoic (linear: *P* < 0.001) acid concentrations as well as total SFAs (linear: *P* < 0.01) and *n*-3 PUFAs (quadratic: *P* < 0.01). It was noted that the *n*-6/*n*-3 PUFA ratio was lowest (3.63) in the meat from birds fed diets with 2.5% OMSS. Notwithstanding, the spent substrate had no effects on all other meat fatty acids (*P >* 0.05).

**Table 6:** Effects of dietary *Vachellia erioloba* pods oyster mushroom spent substrate inclusion on meat physico-chemical quality of Boschveld indigenous chickens.

Parameter	Dietary OMSS inclusion level (%)					SEM	*P*-value	
	0	1.25	2.5	5	10		Linear	Quadratic
pH_45min_	5.87	5.67	5.67	5.81	5.64	0.08	0.311	0.961
Temperature_45min_	23.74	24.50	23.60	24.21	24.19	0.58	0.714	0.931
L*_45min_	53.30	53.88	52.48	53.26	57.04	1.88	0.122	0.319
a*_45min_	0.42	0.78	0.79	0.66	0.37	0.15	0.245	0.066
b*_45min_	1.76	2.52	2.71	2.07	2.71	0.53	0.454	0.844
H_45min_	1.22	1.23	1.20	1.09	1.43	0.15	0.335	0.249
C_45min_	4.17	7.95	10.01	6.73	8.19	2.77	0.599	0.506
pH_24h_	5.96	5.99	6.06	6.10	6.09	0.07	0.173	0.299
Temperature_24h_	11.65	12.19	11.31	11.28	10.26	0.62	0.740	0.758
L*_24 h_	53.48^b^	53.99^ab^	53.52^b^	55.75^a^	58.41^c^	1.17	*P* < 0.01	0.647
a*_24 h_	0.56^b^	0.73^ab^	1.01^a^	0.76^ab^	0.42^b^	0.18	0.265	*P <* 0.05
*b*_24h_	2.18^b^	2.91^ab^	3.81^a^	3.46^ab^	3.73^a^	0.50	*P <* 0.05	0.159
H_24h_	1.28^ab^	1.24^b^	1.23^b^	1.36^ab^	1.46^a^	0.11	0.118	*P <* 0.05
C_24h_	5.66	11.38	17.58	12.93	14.57	3.30	0.197	0.159
WHC (%)	81.31	84.47	83.79	87.41	85.55	2.76	0.241	0.082
Drip loss (%)	4.16^b^	4.40^ab^	4.55^ab^	5.19^a^	4.23^b^	0.53	0.881	*P <* 0.05
Cooking loss (%)	37.09	41.54	38.61	41.30	36.95	2.89	0.702	0.277
Shear force (N)	10.05	18.11	14.45	12.97	13.54	2.21	0.991	0.495

L* lightness, a* redness, b* yellowness, C* chroma, H* hue angle, *WHC* water holding capacity, *OMSS* oyster mushroom spent substrate, *SEM* standard error of the mean.

**Table 7:** Effects of dietary *Vachellia erioloba* pods oyster mushroom spent substrate inclusion on fatty acid composition (g/100 g), totals and ratios of breast meat of Boschveld indigenous chickens.

Parameter	Dietary OMSS inclusion level (%)					SEM	*P*-value	
	0	1.25	2.5	5	10		Linear	Quadratic
*SFAs*								
Myristic acid	0.36^a^	0.27^b^	0.28^b^	0.22^b^	0.22^b^	0.02	*P* < 0.01	*P* < 0.05
Palmitic acid	20.92^a^	19.72^a^	18.86^b^	18.50^b^	19.03^ab^	0.53	*P* < 0.05	0.066
Stearic acid	8.72^b^	10.06^a^	10.53^a^	10.88^a^	10.52^a^	0.28	*P* < 0.001	*P* < 0.01
Tricosanoic acid	6.54^b^	10.74^a^	12.02^a^	12.25^a^	12.53^a^	0.74	*P* < 0.001	*P* < 0.05
Total SFAs	34.83^b^	40.79^a^	41.69^a^	41.61^a^	42.59^a^	1.047	*P* < 0.01	*P* < 0.05
*MUFAs*								
Palmitoleic acid	2.72^a^	1.77^b^	1.21^b^	1.29^b^	1.28^b^	0.23	*P* < 0.01	*P* < 0.05
Oleic acid	28.69^a^	22.09^b^	20.05^b^	21.07^b^	20.52^b^	1.37	*P* < 0.01	0.079
*Cis*-vaccenic acid	2.16	2.32	2.40	2.30	2.60	0.16	0.449	0.834
Total MUFAs	33.54^a^	26.18^b^	23.65^b^	24.34^b^	24.65^b^	1.602	*P* < 0.05	*P* < 0.01
*PUFAs*								
Eicosadienoic acid	0.30	0.35	0.48	0.27	0.41	0.05	0.492	0.777
Linoleic acid	19.86	16.50	15.07	15.71	14.29	1.18	0.169	0.159
α-Linolenic acid	1.21	0.72	0.67	0.74	0.53	0.12	0.163	0.256
Dihomo-α-linolenic	0.47	0.65	0.65	0.51	0.78	0.07	0.439	0.784
Docosahexaenoic acid	1.42^b^	1.78^b^	3.02^a^	2.70^a^	2.22^ab^	0.25	*P* < 0.05	*P* < 0.01
Total PUFAs	23.22^a^	20.01^ab^	19.89^ab^	20.19^ab^	17.51^b^	1.268	*P <* 0.05	0.3762
Omega-3 (*n*-3) PUFAs	3.11^b^	3.15^b^	4.33^a^	3.94^ab^	3.43^b^	0.251	0.4567	*P <* 0.01
Omega-6 (*n*-6) PUFAs	20.11^a^	16.85^ab^	15.56^b^	16.25^b^	14.08^b^	1.181	*P <* 0.05	0.1214
*n*-6/*n*-3	6.77^a^	5.37^ab^	3.63^b^	4.17^b^	4.17^b^	0.514	*P* < 0.05	*P* < 0.01

OMSS = oyster mushroom spent substrate, SEM = standard error of the mean, SFAs = saturated fatty acids, MUFAs = mono-unsaturated fatty acids, PUFAs = poly-unsaturated fatty acids.

## Discussion

This study investigated the utility of *V. erioloba* pods solid-state fermented using oyster mushrooms (OMSS) as a nutraceutical feed additive for Boschveld indigenous chicken diets by determining the birds’ responses to the mushroom by-product in terms of growth performance, carcass traits, visceral organs, haemato-biochemistry, and meat quality including fatty acid composition, totals and ratios. The observed lack of effect of dietary OMSS on most performance, carcass characteristics, haemato-biochemistry, and meat quality attributes of Boschveld chickens attests to the relative nutritional and physiological safety of the spent substrate for the native birds. These results are not unexpected considering the sanitizing (antimicrobial) attributes of many bioactive compounds including polyphenols, polysaccharides, and sterols in OMSS and biodegradative effects of its oxidoreductase enzymes in terms of dietary fibre and noxious phytotoxins that induce antinutritional effects in poultry^38^. They largely concur with our recent findings demonstrating no effect of dietary OMSS on overall BWG as well as carcass characteristics, meat quality, and most haemato-biochemical attributes of grower and finisher broilers^39^. However, they disagree with those of Mhlongo and Mnisi^25^ who found increased FI and BWG in Boschveld roosters fed the spent substrate-supplemented diets.

Notwithstanding, the observed quadratically decreased overall FCE of the native birds in response to dietary OMSS and the diet × week interaction effect on this economic trait suggest antinutritional effects of residual *V. erioloba* pods-derived dietary fibre and phytochemicals, in an age-dependent fashion apparently. Indeed, the spent substrate induced deleterious effects on this parameter in younger (i.e., weeks 5 and 6) and immature (i.e., week 11) rather than older (i.e., weeks 12 to 16) birds. Of course, *V. erioloba* pods contain high levels of fibre and other ANFs predominated by prussic acid-convertible cyanogenic glycosides as well as tannins^40,41^, remnants of which remain in the spent substrate following SSF, as shown in Table 1 and as previously demonstrated^42,43^. Upon incorporation into experimental diets, the residual fibre and ANFs would decrease the FCE of the chickens, as it was previously reported by Langhout^44^ and George and Sese^45^. Their negative effects on FCE would inevitably be more significant in younger and immature chickens with immature digestive systems and relatively fewer and less diverse biodegradative gut microbiota compared to older and mature birds^46–48^. Another possible explanation for the observed OMSS-induced quadratic decrease in bird overall FCE may relate to the spent substrate-induced increase in the calcium and calcium-to-phosphorus ratios in the experimental diets. Indeed, high calcium levels in non-ruminant diets are known to decrease the absorption of phosphorus due to formation of insoluble complexes in the intestinal lumen^49^, leading to decreased FCE^50^. Despite the decremental effects of dietary OMSS on FCE in the current study, it is however noteworthy that the spent substrate did not increase the weight of the gizzard, as has been observed in broiler chickens^26,39,51^. This may be related to the digestive system of the native birds being more adaptable to digesting dietary fibre and phytotoxins compared to broilers^52–54^.

The observed quadratic increase in serum albumin and bilirubin suggests enhanced nutritional and antioxidant status of the native birds in response to dietary OMSS. Synthesised in hepatocyte nuclei and readily excreted into blood plasma^55^, serum albumin constitutes 65% of serum proteins and is the primary systemic carrier for nutrients, bilirubin, and drugs throughout the body^56^ whilst it also serves as a potent antioxidant^55,57^. On the other hand, serum bilirubin serves as a biomarker for the antioxidant and inflammatory status of the organism with its decreased levels indicative of oxidative stress and inflammation^58,59^. These two biomolecules are interrelated as 99.9% of unconjugated systemic bilirubin is bound to albumin^60^. Hence, the parallel increase in their serum levels is not unexpected and suggests that OMSS enhanced the protein nutritional status and antioxidant capacity of the indigenous chickens probably arising from the high spent substrate contents of proteins and amino acids^61,62^. Notwithstanding, there was probably too much *V. erioloba* pods-derived residual fibre and ANFs in 5% and 10% OMSS-containing diets that induced antinutritional effects and thus constrained the bioavailability of spent substrate-derived nutraceuticals possibly leading to some protein undernourishment and oxidative stress in birds fed these diets. Considering the observed similarities in serum alkaline phosphatase activities in chickens across the dietary treatments, we discounted the possibility of the birds having endured any liver injuries. The observed serum albumin and bilirubin levels are within the range of values for similar age of Boschveld birds^25,63,64^.

Meat quality, particularly its colour, water retention capacity and other physico-chemical attributes, is critically important as it influences consumer perceptions of its freshness, wholesomeness, as well as purchasing decisions. The colour of the meat can be influenced by a variety of factors including diet, genotype, pre-slaughter stress, as well as prevailing conditions during slaughter, chilling, and processing^65^. The mechanism underlying the observed linear increases in meat lightness and yellowness contrasting with quadratic increases in its redness and hue angle may be related to the nutraceutical composition of OMSS and redox status of the meat. In this regard, the OMSS contains high concentrations of antioxidant bioactive compounds including polyphenols, aglycone isoflavone, and others^66^, in addition to iron (heme)-containing ligninolytic peroxidases (lignin peroxidase, manganese peroxidase and versatile peroxidase)^38,67^. Evidently, the incremental dietary inclusions of the spent substrate linearly provided antioxidant biomolecules and enzymes that reduced meat metmyoglobin to the purple-reddish coloured deoxymyoglobin leading to lighter meat^68^. Also, the residual *V. erioloba* pods-derived pigments would have been incrementally deposited into the meat rendering it yellowish in colour, as has been shown in meat from birds fed green plant materials^69^. However, as has been discussed above, there was probably too much pods-derived residual ANFs in the diets with high levels (5 and 10%) of OMSS that may have bound the antioxidant dietary nutrients resulting to the birds suffering from some undernutrition-associated oxidative stress. Indeed, protein undernutrition is known to induce oxidative stress and lipid peroxidation in broilers^70^. This would have oxidized the bright, red-coloured meat oxymyoglobin to the brownish metmyoglobin in birds fed high dietary levels of the spent substrate^68^, which would also explain the observed 24 h post-slaughter increases in meat hue angle and drip loss. Certainly, meat oxidation and lipid peroxidation induce meat discoloration and drip losses, among other things^71,72^. Such discoloured meat with poor water retention capacity is undesirable as it is perceived by consumers to lack freshness and wholesomeness^73–75^.

As part of evaluation of meat quality of the native birds, we measured their breast meat fatty acid responses to dietary OMSS. The observed spent substrate-induced linear decrease in chicken meat myristic and palmitic acids is highly desirable as these SFAs exacerbate human obesity-associated insulin resistance and increase the risk of inflammation and cardiovascular disease (CVD)^76,77^. On the other hand, the observation of OMSS-induced increase in meat stearic acid content is undesirable as this SFA is associated with inflammation and CVD^78^. Similarly, the observed dietary spent substrate-induced decrease in meat palmitoleic and oleic acid is undesirable in terms of human health. Palmitoleic acid ameliorates CVD, diabetes, inflammation^79^ and stroke^80^ whilst oleic acid has antilipidemic and anti-atherogenic effects with demonstrated ability to decrease systemic cholesterol particularly its low-density lipoprotein (LDL) whilst increasing its high-density lipoprotein (HDL) species^81–83^. Our results refute those of Mabuza et al.^39^ who found increased broiler chicken breast meat concentrations of myristic, palmitic, palmitoleic, and oleic acids alongside decreased levels of stearic, eicosenoic and eicosadienoic acids in response to dietary feeding of same incremental inclusion levels of OMSS. The variation between these studies in terms of meat fatty acid composition might be due to breed differences in lipid metabolism between broilers and indigenous birds, as has been reported in previous studies^84,85^. Interestingly, however, our results further demonstrated dietary feeding of the spent substrate to have increased the meat content of DHA and tricosanoic acids. A predominant *omega*-3 long-chain PUFA in human brain and eyes, DHA is required for foetal development, prevention of preterm births and CVD, as well as improvements of cognitive function and eye health^86^. Also, tricosanoic acid is positively correlated with fast learning ability and HDL cholesterol whilst negatively correlated with triglycerides^87^. Therefore, the observed OMSS-induced increase in the indigenous Boschveld chicken meat contents of these unsaturated fatty acids in the current study is highly desirable in terms of human health. Unfortunately, there are currently no comparable literature data on Boschveld chicken meat DHA and tricosanoic acid responses to dietary OMSS feeding.

Lastly, the observed OMSS-induced increase in meat total SFAs in contrast to decreased contents of its total MUFAs and PUFAs is undesirable. Meanwhile, the observed spent substrate-induced decrease in meat total *n*-6 PUFAs in contrast to increased contents of its *n*-3 PUFAs is highly desirable. In this regard, SFAs are generally known to elevate human blood cholesterol levels leading to CVDs^88,89^ with health officials worldwide recommending a decrease of SFA intake to 10% of total energy consumption for both children and adults as a strategy to reduce their CVD-linked LDL cholesterol levels^90^. On the other hand, MUFAs and PUFAs are generally beneficial towards cardiovascular health^91^. Notwithstanding, excessive human consumption of *n*-6 PUFAs is undesirable^92^ due to its linkage to high cancer risk^93^ whereas a higher intake of *n*-3 PUFAs desirably decreases breast and colon cancer risk^93^. Hence the need for regulation of human dietary intake of these *omega* fatty acids in order to balance their body contents and prevent coronary heart diseases^92^.

Furthermore, we determined the meat *n*-6/*n*-3 PUFA ratio as an index of the nutritional quality of its fat and its potential for enhancing human health. Compared to *omega*-3 PUFAs, *n*-6 PUFAs particularly linoleic acid are detrimental towards human health due to their propensity to engender cellular and systemic free radical release and consequent oxidative stress, inflammation and increased risk of atherosclerotic CVD^94,95^. Consequently, a high *n*-6/*n*-3 PUFA ratio (10:1 to 20:1) of a food promotes the genesis of multiple diseases whilst a low ratio (≤ 4 : 1) suggests its enhanced healthiness for the human consumer^96,97^. Thus, the observed quadratic OMSS-induced decrease in this ratio in the indigenous chicken meat is highly desirable. Its lowest value (3.63) in meat from the native chickens fed diets incorporated with 2.5% OMSS suggests that these birds produced the healthiest meat with the optimal *n*-6/*n*-3 PUFA ratio (1 to 4 : 1), similarly to that of ancient human diets^96^. Since indigenous chickens are reared and valued primarily as a source of food (meat)^98^, the optimum dietary inclusion level of OMSS that would enhance the nutritional quality and healthiness of their meat would seem to be 2.5%.

## Conclusion

Our study demonstrated that dietary feeding of *V. erioloba* pods-derived OMSS enhanced the nutritional healthiness of meat without majorly altering its physico-chemical quality as well as growth performance, carcass traits, and haemato-biochemistry in Boschveld indigenous chickens. For production of meat that is healthy for human consumption, we recommend incorporation of the spent substrate in indigenous chicken diets at 2.5% inclusion level.

## Data Availability

Correspondence and requests for data supporting this study should be addressed to the corresponding author.
